# Mesenchymal Stem Cell Senescence and Rejuvenation: Current Status and Challenges

**DOI:** 10.3389/fcell.2020.00364

**Published:** 2020-06-03

**Authors:** Xueke Zhou, Yimei Hong, Hao Zhang, Xin Li

**Affiliations:** ^1^Department of Emergency Medicine, Department of Emergency and Critical Care Medicine, Guangdong Provincial People’s Hospital, Guangdong Academy of Medical Sciences, Guangzhou, China; ^2^School of Medicine, South China University of Technology, Guangzhou, China; ^3^School of Pharmacy, Bengbu Medical College, Bengbu, China

**Keywords:** mesenchymal stem cells, senescence, autophagy, mitochondrial, telomere, rejuvenation

## Abstract

Over the past decades, mesenchymal stem cell (MSC)-based therapy has been intensively investigated and shown promising results in the treatment of various diseases due to their easy isolation, multiple lineage differentiation potential and immunomodulatory effects. To date, hundreds of phase I and II clinical trials using MSCs have been completed and many are ongoing. Accumulating evidence has shown that transplanted allogeneic MSCs lose their beneficial effects due to immunorejection. Nevertheless, the function of autologous MSCs is adversely affected by age, a process termed senescence, thus limiting their therapeutic potential. Despite great advances in knowledge, the potential mechanisms underlying MSC senescence are not entirely clear. Understanding the molecular mechanisms that contribute to MSC senescence is crucial when exploring novel strategies to rejuvenate senescent MSCs. In this review, we aim to provide an overview of the biological features of senescent MSCs and the recent progress made regarding the underlying mechanisms including epigenetic changes, autophagy, mitochondrial dysfunction and telomere shortening. We also summarize the current approaches to rejuvenate senescent MSCs including gene modification and pretreatment strategies. Collectively, rejuvenation of senescent MSCs is a promising strategy to enhance the efficacy of autologous MSC-based therapy, especially in elderly patients.

## Introduction

Mesenchymal stem cells (MSCs) are fibroblast-like and plastic adherent with a self-renewal ability and multiple differentiation potential. Previous studies have shown that MSCs had been successfully established from bone marrow, umbilical cord blood, periosteum, and adipose tissue (Musina, 2005; Paniushin, 2006). MSCs have the property of self-renewal and differentiate into multiple cell lineages, such as bone, cartilage, adipose, muscle, tendon, stroma, and neuronal cells (Mareschi, 2006). They are widely used as seed cells for therapeutic applications in tissue engineering and regenerative medicine (Mahla, 2016). Their availability and low immunogenicity hold extensive promise for clinical application (Uccelli, 2008; Madrigal, 2014; Childs, 2018). MSCs have been broadly applied in the treatment of various diseases, including graft-vs.-host disease (GVHD) (Landgraf, 2011), Crohn’s disease (CD) (Bernardo, 2009; Zhang, 2018), diabetes mellitus (DM) (Al Demour, 2018), multiple sclerosis (MS) (Iacobaeus, 2019) and myocardial infarction (MI) (Lunde, 2006; Gyongyosi, 2015) etc. Nonetheless accumulating data have demonstrated that discrepancy in the effects of MSC-based therapy may be due to senescence-induced alterations in their function (Schimke, 2015). The neurorestorative potential of MSCs may be limited in aged patients with stroke who have a limited number of MSCs (Lee, 2010; Bang, 2016). Allogeneic MSCs, chosen as the first choice for elderly population with frailty syndrome in a phase I/II clinical trial, avoids the aging-related aberrant microenvironments of MSCs and inflamed-aging (Golpanian, 2016). The senescent features of MSCs include enlarged and more granular morphology, and a deficient capacity for proliferation (Haynesworth, 1997) and differentiation, and secretion of a variety molecules, referred to as a “senescence-associated secretory phenotype (SASP)” (Watanabe, 2017). In addition, senescence is accompanied by changes to nuclear morphology and formation of a distinct chromatin structure called senescence-associated heterochromatic foci (SAHF) (Noren, 2017). Currently, the senescent states of MSCs have been assessed by measuring senescence-associated-β-galactosidase activity, telomere length, gene expression markers, gene methylation and epigenetic markers (Jones, 2019). The mechanisms underlying MSC senescence have attracted attention since senescent MSCs hamper the rapid development of MSC grafting. Numerous studies have focused on these abnormal changes to MSC morphology and function, cells previously considered to be immortal. The mechanisms that underlie these processes remain unclear.

Senescent MSC are normally divided into different stages. After each replication cycle, the length of telomeres is shortened. Once telomeres become critically short, they trigger senescence. This is called replicative senescence (Ho, 2017). Following this, activation of oncogenes induces MSC senescence. This is termed oncogene-induced senescence (Kosar, 2011). Numerous stress stimuli also trigger senescence, known as stress-induced senescence (Kornienko, 2019). Induction of senescence can be mediated as part of the normal development process by several pathways or pluripotency genes. This is referred to as developmental senescence (Liu, 2015). The restricted therapeutic application of senescent MSCs highlights the importance of rescuing the functions of MSCs, namely rejuvenating MSCs, so they can be used for autologous transplantation. Rescuing the functions of MSCs is vital for their regeneration capacity (Block, 2017). Recent research suggests that cellular senescence is a modifiable risk factor, giving hope for autologous MSCs-based therapy (Stolzing, 2008). *In vitro* culture is essential to acquire an adequate number of MSCs for use in cell therapy. In parallel to this, targeting three intrinsic mechanisms of MSC senescence may help hinder MSC aging. In this review, we focus on the mechanisms that underlie MSC senescence including DNA damage, telomere erosion and mitochondrial dysfunction. We also summarize the current strategies being applied to rejuvenate senescent MSCs and enhance their therapeutic efficacy.

## Characteristics of MSC Senescence

Cellular senescence is defined as a state of permanent cell cycle arrest. Cell cycling is halted and cells no longer replicate and/or divide. In senescent MSCs this results in deficient proliferation and differentiation as well as changes to protein expression and chromosome structure. Senescent MSCs usually show an enlarged, more granular and flat fried egg morphology, with constrained nuclei and granular cytoplasm. They also exhibit a decreased cell colony number (CFU), one of the most convenient predictive indicators of MSC senescence (Stolzing, 2008). In addition, the cell population doubling time (CPDT) is prolonged. This may be due to a prolonged G1/G0 phase of the cell cycle and a significantly decreased S phase (Gaur, 2019).

DNA staining of senescent cells has revealed nuclei with small and distinct spots that contain heterochromatin, called senescence-associated heterochromatic foci (SAHF) (Kosar, 2011). Each spot represents condensed chromatin that is transcriptionally inactive, and expression of some transcription factors around this region have been found to be downregulated, such as E2F family members and cyclin A (Narita, 2003). SAHF can be identified by DAPI staining and the presence of heterochromatin-associated histone markers, and high levels of H3K9me3 and H3K27me3 (Koch, 2013). As inhibitory markers, an increase of H3K9me3 and H3K27me3 in gene promotor leads to decreased gene expression. Formation of SAHF is a complex process. Researchers are particularly interested in how genes are regulated and their expression affected during formation of SAHF.

Epigenetic regulation is always involved in histone modification and cellular senescence can be tracked by epigenetic modifications (Wagner, 2019). DNA methylation is the most promising marker to predict MSC senescence (Wagner, 2017). Age-associated hypomethylation occurs in heterochromatic regions of the genome, interfering with transcription factors such as repetitive elements and transposons or methylated-CpG binding proteins, and leading to silencing of the gene (Easwaran, 2019). Multiple age-related genes decrease during senescence, such as lysine specific demethylases (KDM3a-b, KDM5d, and KDM6a-b) (Gronthos and Cakouros, 2019). During the gradual process of MSC senescence, DNMT1 and DNMT3B have been shown to be downregulated with a consequent decrease in DNA methylation (Childs, 2018). These changes are not universal but occur only with specific genes and histone modifications. Senescence-associated DNA-methylation (SA-DNAm) may therefore be used to monitor cellular senescence (Koch, 2013). In addition, the expression of stemness-associated genes such as Oct4, Nanog and Tert, decreases during MSC senescence. With chromatin immunoprecipitation and whole genome sequencing (ChIP-seq), large samples can be sequenced and the epigenome scanned to map the epigenetic landscape and enable detection of cellular senescence. Multiple proteins that typically change may serve as indicators of senescence. Such changes may be tested in blood and measures taken to prevent aging.

MSCs are known to have differentiation potential for osteogenesis and adipogenesis. This ability is altered in senescent MSCs that are more likely to differentiate toward adipogenesis (Andrzejewska, 2019). Bone-formation markers, such as the activity of alkaline phosphatase (ALP) and the expression of osteocalcin (OC), are downregulated in senescent MSCs during culture with osteogenic medium (Abuna, 2016). This change to MSC differentiation greatly limits their application. It is important to maintain their self-renewal ability and multiple differentiation potential.

Senescent cells tend to potentiate their effects to neighboring cells via paracrine mechanisms. This is known as a senescence-associated secretory phenotype (SASP) (Debacq-Chainiaux, 2009; Sikora, 2016). The SASP factors include interleukin-1 (IL-1), IL-6, IL8, matrix metalloproteinase1 (MMP1), TNF-α and vascular endothelial growth factor (VEGF) and so on (Rodier and Campisi, 2011). Senescent cells can exert certain influence on their microenvironment by their secretome. Microvesicles (MVs), is a key component of the cell secretome, can inhibit the growth of tumor and immunomodulatory regulation (Akyurekli, 2015; Xie, 2016).

MSCs accomplish their functions through the secretion of cytokines and growth factors, which exert paracrine and autocrine functions (Ranganath, 2012). MSCs-derived exosomes (MSCs-EXOs) contain biological active molecules from the MSCs, which can regulate immune responses in the body. Exosomes of MSCs contain cytokines, growth factors, various Mrna, and regulatory miRNA. But senescence greatly alters the composition of them, the micro RNAs in exosomes were largely different. Senescence greatly alters the composition of this secretome and hence impairs one of the key MSC biological functions (Özcan, 2015). A SASP is always evident in oncogene-induced senescence (OIS) and often accompanied by a global change in nuclear architecture. A broad spectrum of secretory factors produced by MSCs, such as cytokines and chemotactic and growth factors, has been studied (Alexander, 2013; Lei, 2017). They sequenced and analyzed SASP of different aging cell types and focus on 138 common canonical pathways. Putting them into four categories, extracellular matrix/cytoskeleton/cell junctions; metabolic processes; ox-redox factors; and regulators of gene expression. What should be noted is that modification of the extracellular environment is one of the main tasks of the senescence secretome and leucocyte extravasation signaling” as an overlapping network that is common among the different senescence secretome. Further research find that there are 11 proteins emerged in senescent MSCs only (Özcan, 2016). The amount of components released from SASP may partly depend on the different types of cell senescence and the microenvironment. It appears that senescent cells are prevented from becoming tumorigenic by switching on SASP (Özcan, 2016). Nonetheless SASP is thought to be partially responsible for the persistent chronic inflammation that contributes to multiple age-related phenotypes (Reitinger, 2015). Changes in functionality can lead to unexpected situations: inflammation may alter tissue microenvironments and attract immune cells, leading to tissue and organ damage and contributing to aging making treatment difficult.

Telomere shortening is believed to be a hallmark of MSC senescence, limiting long-term MSC division that is essential for tissue renewal. For this reason, telomere attrition is defined as a type of DNA damage for cells, also as a response to DNA damage that finally leads to cell cycle arrest and cell senescence. Telomerase, a type of enzyme that brings repeated TTAGGG to the chromosome end, prevents telomere attrition and induces telomere elongation. Overexpression of the enzymatic subunit of telomerase, telomerase reverse transcriptase (TERT), increases median lifespan in mice (Patel, 2016). The ability to detect telomere length may thus hold promise as a biomarker in the assessment of MSC senescence. Valid and reliable techniques to quantify telomere length have attracted much attention in the study of senescence. The use of telomere-based tests for the diagnosis and management of cellular senescence are well-established (Baxter, 2004).

## Different Types of MSC Senescence

**FIGURE 1:**
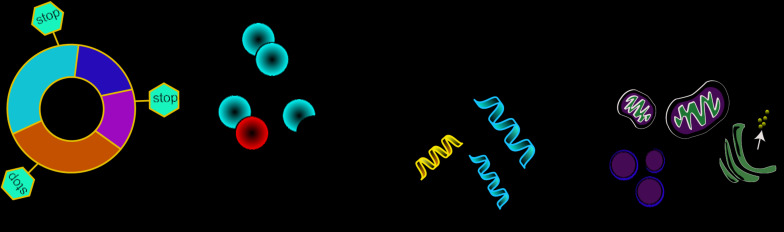
Different types of MSCs senescence.

Normal animal cells undergo senescence after multiple divisions *in vivo* and *in vitro*, and senescent cells will eventually die. Although MSCs have a strong ability to proliferate, they are not infinite. After multiple divisions, cells enter a state of replicating senescence with growth arrest. Studies show that MSCs isolated from elderly individuals have lower proliferation and anti-apoptosis ability than those isolated from young individuals. This is usually referred to as developmental senescence. When stimulated by oxidative stress, MSCs will begin the aging process early, that is, premature aging. This premature senescence may be classified as oncogene-induced senescence or stress-induced senescence. Based on recent published data, we briefly describe the different types of senescence (Figure 1).

## Replicative Senescence

Replicative senescence is defined as an irreversibly restricted proliferation due to telomere erosion in MSCs after a stereotypical number of cell divisions. Nonetheless replicative senescence is also intimately connected to other types of senescence including oncogene-induced (p53 and p16/Rb tumor suppressor pathways etc.), stress-induced (oxidative stress etc.) and even developmental senescence. Ultimately though they are all associated with telomere shortening and consequent replicative senescence. MSCs from elderly individuals recapitulate most parameters seen in senescent MSCs, including a flat, enlarged morphology, a great number of cells staining positive for SA-β-Gal, and lower proliferation rate. These characters have fueled the perception that replicative senescence *in vitro* may serve as a candidate model to unravel the molecular mechanisms that drive the process of body aging. Replicative senescence of MSCs is a continuous process starting from the first passage and there is a dynamic change to senescence-related indicators. Long-term alterations to phenotype, differentiation potential, whole-map gene expression patterns and miRNA profiles are influenced by replicative senescence and all need to be considered as therapeutic targets for MSC rejuvenation (Ho, 2017).

## Oncogene-Induced Senescence

Oncogene-induced senescence (OIS) depends on activation and/or overexpression of oncogenes, such as cyclin E, RAF, MEK, and BRAF. Oncogenic activation has been recognized as a necessary step in tumorigenesis but may also act as a genetic stress and cause irreversible growth arrest in cultured cells. Tumor suppressor genes p16 and p21 play an important role in monitoring the normal integrity of DNA. Senescence of MSCs has been shown to be reversed by ablation of p16 or p21 (Chikenji, 2019). The protein level of p16 or p21 may indicate the parallel level of MSC senescence. For example, knockdown of p16 or p21 in senescent MSCs has been shown to increase their proliferation rate and differential potential (Mas-Bargues, 2017). Signaling pathways, not only individual genes, are always involved in senescence. The mitogen-activated protein kinase (MAPK) pathway can be activated by Ras and plays a role. Two major tumor suppressor pathways, the p14ARF-MDM2-p53 pathway and p16INK4A/pRb pathway, have been shown to be involved in the control of permanent MSC senescence (Clarke, 2004; Liu, 2015; Piccinato, 2015). OIS is often accompanied by a global change in nuclear architecture, most dramatically exemplified by the formation of SAHF. As previously mentioned, heterochromatin-associated histone markers, DNA methylation in particular, is present in SAHF. Gene expression can be regulated by DNA methylation through interference with transcription factors or methyl-CpG binding proteins (Jaenisch and Bird, 2003). Abnormal regulation of methylation will lead to the disorder of replication in cell, which resulting in DNA replication errors, thereby induce cell apoptosis.

In contrast to hyper-methylation that suppresses the translation of genes, hypomethylation enables genes to be “released” and start replication and translation. These abnormally expressed proteins trigger an intracellular response, much like hyperexpression of Ras in mammal MSCs triggers activation of tumor suppression pathways, and thus induces irreversible growth arrest (Moumtzi, 2010). In the presence of the hyperproliferative signals during the process of senescence, cells encounter a strong DNA replication stress and finally develop numerous double-stranded DNA breaks (DSBs) in fragile areas of DNA (Hladik, 2019). The damaged DNA released from the nucleus may activate inflammatory pathways and eventually lead to apoptosis. On the contrary, the accumulation of tumor suppressor gene products caused by the abnormal mitosis suggests that OIS is an anti-tumor reaction that can ensure cell proliferation within an allowable range.

## Stress-Induced Senescence

Stress-induced premature senescence (SIPS) occurs as a result of many different stimulations including reactive oxygen species (ROS), ionizing radiation, osmotic stress, mechanical stress, hypoxia, and heat shock (Zglinicki, 2000). There are numerous cellular and molecular features that are similar for cells with SIPS and those undergoing replicative senescence although they occur at the stages of senescence. The mechanisms that underlie SIPS, especially ROS production by damaged mitochondria, involve many signaling pathways. ROS is an important factor during senescence and has been extensively studied. Indeed, our previous studies also showed that ROS plays a critical role in regulating MSC senescence (Huang, 2019; Li, 2019). An imbalance of ROS and anti-oxidants such as superoxide dismutase (SOD) in senescent MSCs initiates growth arrest, regulated by intricate networks of molecular signaling pathways (Bi, 2018). FOXO, whose subfamily (FOXO1, FOXO13a, FOXO14, and FOXO16) is the downstream target of the PI3K-AKT signaling pathway, is another molecule that regulates the ROS pathway during cellular senescence (Fukada, 2014). The p53/p21 pathways and p38MAPK pathways are responsible for the irreversible cell cycle arrest that occurs when MSCs are exposed to ROS, although inhibition of the p38MAPK pathway can restore cell proliferation. Thus controlling ROS may directly alleviate cell senescence.

As another key cause of senescence, DNA damage also plays an important role in activating the p53 pathway to cause cell cycle arrest (Pelicci, 2004). Multiple factors are involved in the repair of damaged DNA. During the replication or repair of damaged DNA, any small accidents can result in large changes: gene editing *in vitro* is one example of a means by which to alter cell phenotype. Various transcription factors including P53 are also recruited by autophagy related protein (ATG), and strengthen the autophagy when stimulated. Aging as a consequence of autophagy has been linked to cellular senescence and autophagy is recognized as a sensor of stress, similar to oxidative stress. Studies have described a decline in autophagy activity and a reduction of autophagy related genes such as Atg1, Atg5, and Atg12 in response to cellular senescence (Fafian-Labora, 2019). The autophagy response is a useful weapon for cells, but the imbalance in autophagy is a threat to their survival.

On the other hand, proteasomes are inhibited by severe oxidative stress. Damage to the proteasome leads to aberrant folding of proteins, toxic aggregation, and accumulation of damaged proteins, further promoting cell senescence. Misfolding or false modification of proteins may cause altered function that in turn leads to abnormal regulation of genes. The synthesis, modification and explanation of proteins has always been a popular subject of research. Due to the strict requirements of the physiological environment for proteins *in vitro*, it is difficult to replicate this *in vivo* synthesis and this is a major obstacle for research.

## Developmental Senescence

Senescence can be induced by regulation of multiple pathways or pluripotent genes in non-pathological states and is a part of normal cellular development. Three signal pathways, insulin-like signaling pathway, target of rapamycin and Sirtuins/NAD +, have been shown to play a major role in MSC senescence (Severino et al., 2013; Gharibi, 2014; Oh, 2014; Chen, 2017). Interestingly, they are all intimately related to metabolism. IGF-1 and insulin signaling, named the “insulin and IGF-1 signaling pathway” (IIS signaling), is a highly conserved signaling pathway that controls aging (Chen, 2016, 2017). Current evidence indicates that IIS signaling plays a key role in regulating aging and longevity (Campisi, 2019). In mice, selective disruption of insulin receptors in adipose tissue extended longevity. Increased lifespan has also been reported in mice with deletion of insulin receptor substrate 1 (IRS1) in whole body or IRS2 only in the brain. Although dietary restriction promotes the proliferation of MSCs, the underlying mechanism may be linked to the pathway.

The mTOR pathway that comprises mTOR complex 1 (mTORC1) and mTOR complex 2 (mTORC2) promotes substance metabolism, participates in cell apoptosis and autophagy, and plays an important role in many diseases. The mTORC1 signaling pathway integrates at least five major intracellular and extracellular signals – growth factors, stress, energy states, oxygen supply, and amino acids – to control processes such as protein-lipid synthesis and autophagy. Studies have shown that the mTORC1 signaling pathway exhibits a pattern of diurnal oscillation. Per2, the core clock protein, can specifically bind to mTORC1 and recruit Tsc1 to mTORC1 as a scaffold protein, thus specifically inhibiting the activity of mTORC1. Activation of mTORC1 is highly associated with a calcifying phenotype of MSCs. Transition from stemness one to osteoblast remarks possibly cellular senescence in MSCs. However, reciprocal activation of mTORC2 protects MSCs from calcification to promotes protective cell fates (Gharibi, 2014; Zhang, 2017; Yang, 2018; Schaub, 2019; Wu, 2019).

Sirt1 (Sirtuin type1), a member of the Sirtuins family, is a histone deacetylase that is dependent on nicotinamide adenine dinucleotide (NAD +), and deacetylation of several transcription factors that control metabolic and endocrine signals regulates its activity *in vivo* (Yuan, 2012; Pi, 2019). It is widely involved in the regulation of mammalian cell life signals, glucose metabolism, insulin secretion and other metabolic pathways, and plays an important role in metabolic syndrome, cell apoptosis, cardiovascular diseases and neurodegenerative diseases. Sirt1 has been increasingly valued as a therapeutic target for many diseases. Reduced expression of Sirt1 impairs the adipocyte differentiation ability of MSCs (Khanh, 2018); overexpression of Sirt1 reduces the acetylation of Bmi1, which is tightly correlated with MSCs osteogenic ability (Wang, 2019).

As relatively complex and powerful signaling pathways, these three pathways play an important role in cell development. Research on them ongoing, and more mechanisms will be discovered that may extend cell life or slow cell aging.

## Mechanisms of MSC Senescence

The mechanisms involved in different types of aging are not entirely consistent, but most are interrelated and interact with each other. These four mechanisms, particularly DNA damage, mitochondria and autophagy, are closely related and play a crucial role in stem cell senescence (Li, 2017). The following is a brief discussion based on the available data.

## DNA Damage

DNA, as the most important genetic material of an organism, can maintain its own stability. DNA damage can accelerate cell senescence and apoptosis, and cause diseases such as cancer and tumors. MSCs are prone to DNA damage during proliferation. When DNA damage reaches a certain level, abnormal cell cycling may ensue (Hladik, 2019). Testing with antibodies that recognize the phosphorylated form of histone H2AX (γH2AX) (Kozlowski, 2015), a histone variant of the H2A protein family phosphorylated rapidly following DNA damage, has been used to assess DNA damage (Gaur, 2019).

Oxidative stress is considered the main cause of DNA damage and aging, and the occurrence of cellular senescence is closely related to reactive oxygen species (ROS). Data show that sublethal ROS and ionizing radiation can cause DNA damage to MSCs derived from human umbilical cord. The increased intracellular ROS is an important cause of senescence of bone marrow-MSCs (BM-MSCs) that show reduced DNA synthesis and cell proliferation and consequent cell senescence (Chen, 2019). Cells cultured *in vitro* in a high oxygen environment show accumulation of ROS in cells with consequent activation of the stress signaling pathway and cell senescence due to oxidative stress (Infante and Rodríguez, 2018). ROS accumulates during normal cell metabolism and a low concentration is essential for cell proliferation and differentiation. Nonetheless a high level of ROS is produced in pathological conditions. High concentrations of ROS have a strong cytotoxic effect and induce cell damage (Kozlowski, 2015). Studies have shown that ROS is significantly increased in apoptotic cells compared with normal cells. The increase in ROS production by senescent MSCs results in excessive ROS or exogenous H_2_O_2_ that can impair proliferation and differentiation of MSCs (Jeong and Cho, 2015). Increased ROS can induce MSC senescence which can be partially reversed by N-acetylcysteine, an oxygen scavenger, with consequent reduction in DNA damage (Zhang, 2013). Thus, increasing the activity of DNA repair pathways may aid recovery of senescent MSCs.

## Telomere Erosion

The telomere is a special structure located at the end of linear chromosomes in eukaryotic cells. Each time the DNA replicates, the telomere is shortened. Telomere shortening is one of the endogenous changes that occur in MSCs during aging. As MSCs passage, telomeres will gradually shorten. When telomeres are shortened such that DNA replication can no longer continue and chromosomal stability cannot be guaranteed, senescence will ensue (Montpetit, 2014; Lai, 2018). Telomere length is mainly maintained by telomerase. Inhibiting the expression of SIRT1 in liver cancer cells has been shown to decrease the expression of telomerase and cause the telomere to shorten with consequent induction of cell senescence or apoptosis (Yamashita, 2012). Disorders of telomere function can also occur in some diseases. Bone marrow mesenchymal stem cells (BMSCs) derived from congenital dyskeratosis show reduced colony formation and differentiate into adipocytes and fibroblasts spontaneously, and show signs of senescence. Gene-related telomere mutations that cause shortening of telomeres are the main cause of this disease (Nadeau, 2019). Nonetheless other studies have reported that knockout of SIRT1 in BMSCs resulted in slower cell growth and accelerated cell senescence, while overexpression of SIRT1 delayed the senescence and maintained the potential for osteogenic and lipogenic differentiation (Chen, 2014). Additionally, overexpression of human telomerase reverse transcriptase (hTERT) can activate telomerase activity and maintain telomere length. Data showed less damage due to external oxidative insult in the nuclei of hTERT-overexpressing cells compared with the control cells (Trachana, 2017).

Nonetheless the level of telomerase in cells is almost undetectable, and overexpression of telomerase can prolong telomeres. It is also unknown whether the introduction of viral plasmids will cause safety issues. Moreover, the large-scale telomere prolongation will cause some cells to lose control with a subsequent risk of tumorigenesis. Therefore, targeted regulation of telomeres in specific cells is also a prospect for future therapies, similar to CAR-T treatment in leukemia. Scientists are also trying to explore ways to reprogram *in vivo*, to ensure safer treatment.

## Mitochondrial Dysfunction

Mitochondria are central to cellular respiration and involved in various cellular activities such as cell matrix metabolism, apoptosis, and initiation of signal transduction pathways. Reductions in mitochondrial function and consequent respiratory chain dysfunction have been observed in senescent MSCs (Lonergan, 2006). Under normal circumstances, mitochondrial fission produces small round mitochondria and generates chain-like mitochondrial tubules. Once suffered from external serious attack, mitochondrial fission will occur and dysfunctional mitochondrion will be cleared by mitochondrial autophagy. Defects in mitochondrial function such as reduced membrane potential, open mitochondria permeability transition pore (mPTP), or increased oxidative stress will eventually lead to apoptosis or cell death. The disturbed mitochondrial dynamics that occurs in cellular senescence affects morphology of MSCs (Herranz and Gil, 2018). In replicative senescence, MSCs enter a normal senescent stage with elongated mitochondrion and damaged function. MSCs that suffer an external serious attack have discrete, fragmentary mitochondrion.

The accumulation of ROS in mitochondria is the main cause of mitochondrial dysfunction. In turn, damaged mitochondria produce more ROS. Mitochondrial oxygen consumption decreases in the later passage of MSCs indicating that cell senescence depends on the accumulation of ROS. At the same time, ROS is an important factor that affects MSC senescence (Sahin and Depinho, 2010; Ghanta, 2017). Mitochondria, as the energy centers in cells, are involved in many cell activities and a decline in mitochondrial function plays a role in aging in humans. It has been reported that a previously infertile woman gave birth following mitochondrial transplantation. Similarly, mitochondrial transplantation provides a solution for aging cells. Nonetheless many issues remain with regards mitochondrial treatment and further exploration is required.

## Autophagy Imbalance

Autophagy is a highly conserved physiological process that is widespread in eukaryotic cells (Revuelta, 2017). It plays an important role in maintaining bio-energetic homeostasis through the control of molecular degradation and organelle turnover (Eckhart, 2019), but excessive autophagy can lead to cell death (Mortensen, 2011). When cells are exposed to internal and external stress (such as oxidative stress, hypoxia, and nutritional deficiencies), cell autophagy will be strengthened. Activated autophagy constitutes a stress adaptation pathway that promotes cell health and survival, and prevents the accumulation of detrimental components that could result in cell damage and death (Matheu, 2017). Autophagy gradually loses its function with the growth of age, efficiency also decreases (Rubinsztein, 2011). On the contrary, enhancing autophagy function can prolong the life of organisms. Therefore, autophagy can improve protein homeostasis and mitochondrial homeostasis, delaying organ function degradation and achieving life extension (López-Otín, 2013). Previous researches showed that inhibition of autophagy can reduce cell senescence induced by proto-oncogene activation (Young, 2009). Dysfunctional proteins and damaged organelles accumulate during cellular senescence and Autophagy can removes aged or damaged organelles and ensures the normal turnover of long-living proteins.

Autophagy is also necessary for the proliferation and differentiation of MSCs. Downregulation of autophagy can limit the therapeutic actions of MSCs (Ma, 2018). Various stresses can induce autophagy of MSCs. For example, oxidative stress can induce cell apoptosis while promoting autophagy. Autophagy is closely related to senescence of MSCs with levels increased during replicative senescence or induced senescence. An increased level of autophagy has been detected in MSCs treated with glucose at high concentrations *in vitro* (Stolzing et al., 2006). The amount of ROS also increases in replicative senescence, and senescence of MSCs can be alleviated by down-regulating autophagy levels (Infante, 2014). Consistently, adipose-derived MSCs isolated from patients with abdominal aortic aneurysm exhibit senescence phenomena that increased SASP and decreased proliferation. Treatment of these MSCs with rapamycin (an autophagy activator) remarkably downregulated SASP (Oxid Med Cell Longev. 2019 Nov 25; 2019:1305049). These findings suggest that regulating the autophagy level is a novel strategy to rejuvenate senescent MSCs.

## Rejuvenation of Senescent MSCs

Auto-transplantation of MSCs has been shown to improve the function of MSCs extracted from patients (Sun, 2019). The same is urgently needed to improve MSC performance *in vitro*. Rejuvenation of MSCs is broadly defined as a reversal to the embryonic state or a slowing down of the aging process. In general, approaches to achieve this have been based on genetic modification, microRNA treatment and preconditioning modification (Neves, 2017; Ocansey, 2020).

## MSCs Reprogramming

**FIGURE 2:**
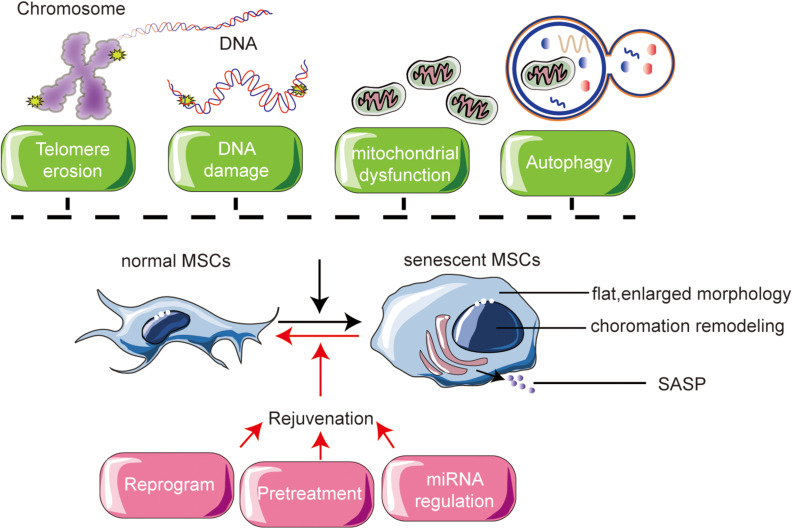
Mechanisms of MSC senescence and their rejuvenation.

Two types of reprogramming are involved in modifying MSCs (Figure 2). Fully reprogramming refers to resetting of epigenetic clocks by reprogramming within iPSCs (Galkin, 2019). Partial reprogramming is similarly regarded as epigenetic rejuvenation and involves DNA methylation and histone modification (Ocampo, 2016).

First reported in 2006, induced pluripotent stem cells (iPSCs) have been established as useful tools for regenerative medicine (Marion, 2009; Frobel, 2014). Functional MSCs have been successfully induced from iPSCs, named induced MSCs (iMSCs) (Hynes, 2013), and been shown to have improved cell vitality. iMSCs generated from iPSCs show typical characteristics of MSCs, but little epigenetic change. Compared with adult MSCs and irrespective of donor age or cell source, iMSCs show a rejuvenated profile (Spitzhorn, 2019). Nonetheless DNA methylation, related to age, was completely erased, and iMSCs reacquired senescence-associated DNA methylation during culture *in vitro*. Interestingly, overexpression of pluripotency factors without reprogramming failed to ameliorate molecular and epigenetic hallmarks of senescence (Gobel, 2018). In addition to four established factors OSKM (Oct4, Sox2, Klf4, and c-Myc), work on reprogramming with three factors, seven factors or chemical factors is ongoing. Nonetheless the efficiency is low, the number of cells available is limited, and the cost is high. There remains a large gap between the laboratory and the bedside. At present, this technology provides us with a means to study the mechanisms of aging and may at some point help prevent or treat aging.

It is proposed that partial reprogramming enables the generation of rejuvenated cells without having to go through a dedifferentiation cycle. Both hypomethylation and hypermethylation are found in aged MSCs. MSCs acquire continuous changes in gene expression and DNA methylation over subsequent passages, these senescence-associated (SA) modifications even be used as biomarker to account for the number of passages or the time of *in vitro* culture (Koch, 2012; Schellenberg, 2014; Fernandez-Rebollo, 2020). As previously noted, almost one third of the CpG sites reveal age-associated changes on DNA methylation, of which 60% become hypomethylated and 40% hypermethylated upon aging. Several researches aimed to investigate epigenetic modulation of senescent MSCs.

First, gene expression can be regulated by DNA methylation through silencing of respective promoter regions. 5-Azacytidine (5-AZA), an inhibitor of DNA methyltransferase (DNMT), reverses the aged phenotype of MSCs via reduction of reactive oxygen ROS accumulation, amelioration of superoxide dismutase activity and increased BCL-2/BAX ratio (Kornicka, 2017). The DNA methyltransferase inhibitor RG108 significantly induces expression of TERT by blocking methylation at the TERT promoter region. DNMT1 and DNMT3B, belong to DNA methyltransferases (DNMTs) which modulate the patterns of polycomb-mediated histone methylation, are significantly decreased during the replicative senescence of MSCs. In contrast, expression of DNMT3a was found to be increased during replicative senescence, participating in the new methylation associated with senescence (So, 2011). Therefore, hypomethylation is evident in aged MSCs, while demethylation at the promoter region of irreplaceable protein plays an important part in maintaining MSC phenotype, lifecycle elongation and regeneration.

Second, modification of histone has attracted attention in epigenetic modulation of senescent MSCs. It has been demonstrated that tetramethylpyrazine (TMP) significantly inhibits the cell senescent phenotype by modulating EZH2 (a histone-lysine N-methyltransferase enzyme)-H3k27me3, suggesting tri-methylation at the 27th lysine residue of the histone H3 protein (Gao, 2018). Restoring mitochondrial NAD + levels by overexpressing NNT and NMNAT3 and delaying replicative senescence can enhance reprogramming efficiency of aged MSCs (Son, 2016).

It is established that telomere shortening due to telomerase deficiency leads to progressive senescence of MSCs. Approaches to transiently enhance telomerase activity are required in order to rejuvenate MSCs. Overexpression of the catalytic subunit of the human telomerase (TERT) results in telomere extension, but does not prevent senescence-associated DNA methylation (Wagner, 2017). Previous work has shown that pretreatment with MIF improved the telomerase activity of MSCs via the PI3K-Akt signaling pathway (Xia and Hou, 2018).

## Micro RNA Treatment

It is becoming increasingly clear that in addition to coding genes, non-coding RNAs also regulate gene expression (Abdelmohsen, 2015). A summary of studies of miRNA and the function of microRNA in retraining MSCs from senescence is shown in Table 1. MicroRNA-based treatments show multidimensional targets and delayed MSC senescence. However, one or two microRNAs in the therapy of senescent MSCs appears to have little effect. Mixing several senescent-associated microRNAs together to determine the efficiency of treatments may be a new objective.

**TABLE 1:** Summary of published data on the application of microRNA retraining of MSCs from senescence.

**MicroRNA**	**Targeted cell**	**Mechanism**	**Rejuvenation of function**	**References**
miR-217 overexpression	BMMSCs	Targeted to DKK1	Osteogenic differentiation	Dai, 2019
Downregulation of miR-196	BMMSCs	Targeted to HOXB7	An improved osteogenesis	Candini, 2015
Downregulation of miR−195	BMMSCs	Targeted to Tert	Reactivating telomerase	Okada, 2016
Downregulation of miR-34a	BMMSCs	Targets SIRT1	Activation of the SIRT1/FOXO3a pathway, improve mitochondrial function	Zhang, 2015
Downregulation of miR-29b-3p	BMMSCs	Targets SIRT1	Regulates aging-associated insulin resistance	Su, 2019
Downregulation of miR-29c-3p	BMMSCs	Targets CNOT6	Affected the p53–p21 and the p16–pRB pathways	Shang, 2016
Downregulation of miR-27b	Ad-MSCs	Unknown	Downregulated p16 and MARP3 genes, increased MSC migration	Meng, 2018
miR-211 overexpression	BMMSCs	Targets STAT5A	Enhance migration ability	Hu, 2016
Downregulation of miR-141-3p	UCB-MSCs	Targets ZMPSTE24	Suppression of an abnormal nuclear phenotype in the HDAC-inhibitor-treated cells	Yu, 2014
Upregulation of miR-10b	Ad-MSCs	Targets SMAD2	A balancing osteogenic and adipogenic differentiation	Li, 2018
Upregulation of miR-10a	BMMSCs	Targets KLF4	Promoted implanted stem cell survival	Dong, 2018
Downregulation of miR-1292	Ad-MSCs	Targets FZD4	Delay senescence and enhance bone formation	Fan, 2018
Downregulation of miR-31	Ad-MSCs	Targets Frizzled-3	Osteogenesis	Weilner, 2016

## Preconditioning Modification

Data show that ROS increase in aged MSCs and accumulated oxidative damage leads to abnormal proliferation and ultimately MSC senescence. Several studies have shown that MSC senescence may be reversed by modulation of ROS aggregation and oxidative metabolism. Ascorbic acid has been shown to inhibit the production of ROS due to D-galactose and activation of AKT/mTOR signaling in MSCs (Yang, 2018). Other work has revealed that lactoferrin inhibits the production of ROS induced by hydrogen peroxide, and downregulated caspase-3 and AKT activation to reduce hydrogen peroxide-induced apoptosis (Park, 2017). MSCs pretreated with Cirsium setidens, a kind of antioxidant, could inhibit production of ROS and decrease the expression of phosphorylated-p38 mitogen activated protein kinase, c-Jun N-terminal kinase and p53 (Lee, 2016). Overall, controlling ROS at a reasonable level can greatly alleviate cell aging. Nonetheless since many stimuli can increase ROS, it is unknown whether a different drug is needed for each stimulus to achieve down-regulation of ROS. Interestingly, it has been shown that when high doses of antioxidant are applied to proliferating cells to maintain physiological levels of ROS, it can also cause DNA damage and induce premature senescence (Kornienko, 2019). This suggests a need to re-evaluate unconditional anti-aging antioxidant properties.

A combination of mitochondrial biogenesis, mitochondrial dynamics and mitophagy determine mitochondrial morphology and mitochondrial function. Deficient mitochondrial function is often regarded as a typical phenotype of senescent MSCs. Melatonin can rescue MSC senescence by enhancing mitophagy and mitochondrial function through upregulation of heat shock 70 kDa protein 1L (HSPA1L). HSPA1L binds to COX4IA, the mitochondrial complex IV protein, leading to an increase in mitochondrial membrane potential and anti-oxidant enzyme activity (Lee, 2020). The decrease in CPT1A (carnitine palmitoyltransferase1A) reverses mitochondrial dysfunction (decreased ROS and improved mitochondrial membrane potential), and reverses senescence of PD-MSCs (Seok, 2020). Our previous study showed that elevation of FGF21 could improve mitochondrial function to rejuvenate senescent MSCs by regulating mitochondrial dynamics (Li, 2019).

Therefore, optimizing the function of damaged mitochondria is a reliable way to rejuvenate senescence.

Proteostasis is protein homeostasis and involves a highly complex interconnection of pathways that determine the synthesis and degradation of protein. Maintenance of the balance of these processes within an organism is dependent on ubiquitination and autophagy. Protein synthesis is strictly regulated within the cell, and the involvement of transcription factors will affect protein synthesis. FOX is a transcription factor and FOXP1 attenuates aging by directly regulating p16INK4A transcription in MSCs. Overexpression of YAP or FOXD1 rejuvenates aged MSCs. This occurs through overexpression of YAP or FOXD1 that enhances the expression of proliferation markers and genes related to chondrocyte differentiation (Fu, 2019). Histone modification of genes also regulates their expression and thus affects protein synthesis through processes such as DNA methylation and acetylation.

Autophagy has been widely employed as an anti-aging target. Inhibition of mTORC1 with AICAR and NAM boosts autophagy and retains MSC capacity for self-renewal and differentiation, and postpones senescence-associated changes (Khorraminejad-Shirazi, 2020). Hyperactivation of mTOR can negatively regulate autophagy and cause imbalance in the proteasome, ultimately leading to cellular damage and senescence. A molecular link between age-related changes in BMMSCs and autophagy has been demonstrated: expression of p53 and ROS increased in the 3-MA (the autophagy inhibitor)-treated group and decreased in the rapamycin (the autophagy inhibitor)-treated group. AhR inhibition restored autophagy suppressed by kynurenine and increased the expression of senescence associated β-galactosidase and p21, as well as blocking aggregation of nuclear H3K9me3 (Kondrikov, 2020). HIF1α−Notch3−mediated AIMP3 regulation is a key pathway for developing antiaging interventions. Downregulation of AIMP3 (aminoacyl−tRNA synthetase−interacting multifunctional protein 3) ameliorated senescence by activating autophagy in MSCs (Kim, 2019). These results suggest that down-regulation of autophagy can indeed alleviate aging. As mentioned above, autophagy involves many proteins so its control requires the identification of specific mechanisms to enable targeted regulation.

It is well known that phosphatidylinositol 3-kinase (PI3K)/AKT is associated with premature cellular senescence (Gharibi, 2014; Liang, 2019) and scientists have devoted themselves to exploring mechanisms to rescue senescence. FGF-2 appears to maintain MSC stemness by inhibiting cellular senescence through a PI3K/AKT-MDM2 pathway (Coutu, 2011; Matsuda, 2018). Embryonic stem cell-derived extracellular vesicles (ES-EVs) can be used as a pretreatment factor to enhance the therapeutic effect of MSCs, mediated by the IGF1/PI3K/AKT signaling pathway (Zhang, 2019). Inhibition of PI3K/AKT/mTOR significantly increases the expression of some pluripotency genes like NANOG and OCT4 (Lu, 2019). NANOG has been shown effectively to reverse MSC senescence in numerous studies (Mistriotis, 2017). Various underlying mechanisms have been proposed. NANOG upregulates PBX1 (a homeodomain transcription factor) and activates the AKT signaling pathway. A feedback loop likely exists between PBX1 and AKT signaling, maintaining HF-MSCs in a highly proliferative state with differentiation potential (Liu, 2019). NANOG also restores expression of COL3 and thus stabilizes extracellular matrix synthesis (Rong, 2019).

It should be noted that the principle parts of cellular signaling pathways to rescue MSC senescence such as those of AMPK, Sirt1 and FOX, are intimately related to calorie restriction (CR) (Khorraminejad-Shirazi, 2018). CR is often recognized as a vital intervention to prevent or alleviate the severity of aging phenotypes. With CR, the function of senescent MSCs can be enhanced and repaired. CR modulates mitochondrial function and autophagy, eliminating ROS and DNA damage. The most recent research concludes that CR plays a regulatory role in the immune system (Ren, 2017).

## Conclusion

Most cell regulatory processes are not independent events, nor are their effects (Figure 2). MSC senescence is a complex and comprehensive problem, so multiple different approaches are required to alleviate or prevent senescence and improve the clinical application of MSCs. A thorough understanding of the characteristics of MSC senescence, the underlying mechanisms, and different types of senescent MSCs will aid in the search for methods to rejuvenate senescent MSCs. MSCs offer great hope in regenerative medicine. To fulfill their potential, there is an urgent need to understand rejuvenation processes to optimize their application. Fully reprogram and partially reprogram of MSCs are thought to fully or partially erases the transcriptomic signatures of aging present in senescent MSCs. Preconditioning modification of MSCs also recognized as a possible source of patient-specific cells for transplantation therapies.

However, several limitations restrain the application of rejuvenated MSCs from bench to bedside. First, the proliferation arrest is continuously acquired with increasing passages *in vitro* cultivation of MSCs. Then, genetic modification of MSCs possibly end up as a secondary damage. The key pathways regulating senescence of MSCs also are the important physiological regulators of normal biological functions. Therefore, complete suppression or activation these pathways by interventions may be unacceptable. Thus, we have to weigh against the possible side effects and the therapeutic efficiency.

## Author Contributions

XZ and YH searched the literature and wrote the manuscript. HZ searched the literature and provided comments. XL designed the study and wrote the manuscript.

## Conflict of Interest

The authors declare that the research was conducted in the absence of any commercial or financial relationships that could be construed as a potential conflict of interest.
